# Supplementary material for: Osteoarthritis-Induced Metabolic Alterations of Human Hip Chondrocytes
Source: Biomedicines. 2022 Jun 8;10(6):1349. doi: 10.3390/biomedicines10061349 (PMC9220245; doi:10.3390/biomedicines10061349)
Supplement: Supplementary file 1 [file biomedicines-10-01349-s001.zip › biomedicines-1713792-supplementary.pdf]

Supplementary materials

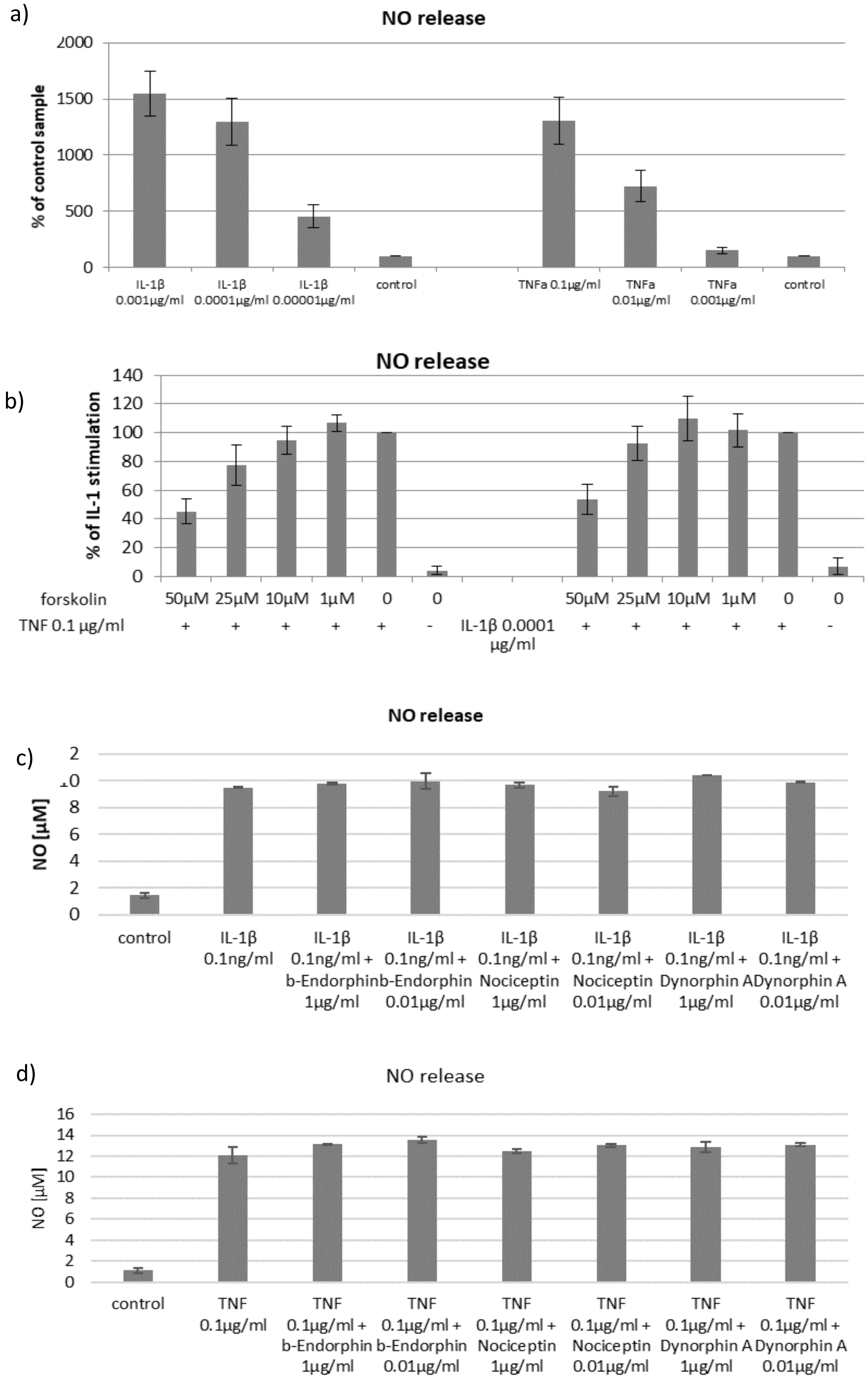

Suppl. Figure S1. Preliminary experiments to evaluated concentrations of a) IL-1β and TNF, b) forskolin, c) and d) opioid neuropeptides for stimulation experiments on human OA chondrocytes. Following concentrations were selected: 0.1 ng/mL IL-1β, 0.1 µg/mL TNF, 50 µM forskolin and 0.1 µg/mL β-endorphin, nociception and dynorphin A.
